# Superoxide dismutase ameliorates oxidative stress and regulates liver transcriptomics to provide therapeutic benefits in hepatic inflammation

**DOI:** 10.7717/peerj.15829

**Published:** 2023-08-11

**Authors:** Longyan Chen, Yang Liu, Yonggang Zhang, Yanmin Zhang, Wei Wang, Hongyu Han, Chunyu Yang, Xueqian Dong

**Affiliations:** 1 Qilu Hospital of Shandong University, Jinan, China; 2 QiLu University of Technology, Jinan, China; 3 State Key Laboratory of Microbial Technology, Shandong University, Qingdao, China

**Keywords:** Superoxide dismutase, Oxidative stress, Hepatic inflammation, Transcriptomics, Deoxynivalenol

## Abstract

**Background:**

Oxidative stress refers to the imbalance between oxidants and antioxidants in organisms and often induces hepatic inflammation. Supplementing exogenous superoxide dismutase is an effective way to alleviate oxidative stress; however, the effects and mechanisms by which superoxide dismutase alleviates hepatic inflammation remain unclear.

**Methods:**

This study established a Kunming mouse model to verify and investigate the oxidative stress and hepatic inflammation-alleviating effects of the superoxide dismutase oral supplement that was prepared by our research group in a previous study.

**Results:**

The superoxide dismutase product significantly restored the body weight and liver alanine transaminase, aspartate aminotransferase, superoxide dismutase, catalase, glutathione, and glutathione peroxidase levels of oxidative stress induced mice. Moreover, exogenous superoxide dismutase significantly inhibited interleukin 1*β* and interleukin 6 mRNA expression in the livers of mice with hepatic inflammation. Transcriptomic analysis indicated that superoxide dismutase had a significant inhibitory effect on *Endog* expression, alleviating oxidative stress damage, and mediating liver cell apoptosis by regulating the expression of *Rab5if*, *Hnrnpab*, and *Ifit1*.

**Conclusion:**

Our research verified the oxidative stress remediation effects of superoxide dismutase and its therapeutic role against hepatic inflammation. This study can lay a foundation for investigating the mechanism by which superoxide dismutase alleviates hepatic disease.

## Introduction

Oxidative stress (OS), which refers to the imbalance between oxidants and antioxidants in an organism, plays a crucial role in the development of several diseases (Singh et al., 2019). Reactive oxygen species (ROS) are markers of OS; however, in living organisms, ROS are attributed to both beneficial and harmful effects. However, dietary and environmental factors could increase ROS content in the body, which can cause OS. Among the various organs, the liver is the most susceptible organ to ROS (Sánchez-Valle et al., 2012). ROS-induced OS leads to the oxidation of biomolecules, such as DNA, lipids, and proteins (Arrigo et al., 2015), thereby regulating protein expression and apoptosis (Li et al., 2015), changing the physiological functions of the liver. Multiple studies have suggested a correlation between OS and hepatic inflammation (Arrigo et al., 2015; Ávila Escalante et al., 2020; Tan, Norhaizan & Liew, 2018). Peroxidation caused by OS can promote apoptosis and increase the expression of interleukin (IL)-6 (Tan, Norhaizan & Liew, 2018), and regulate the expression of nuclear factor *κ*B, activator protein 1, and early growth response factor 1 (Ávila Escalante et al., 2020), to cause inflammation. Conversely, inflammation can also increase ROS production (Alfadda & Sallam, 2012), resulting in a vicious circle between OS and inflammation.

In organisms, several defense mechanisms can actively alleviate ROS-induced OS. Superoxide dismutase (SOD) is a metalloenzyme with well-known antioxidant properties. Exogenous SOD supplementation is an effective way to alleviate OS (Griess et al., 2017). Related research has indicated that based on the alternate reduction-oxidation of metal ions, SOD can effectively catalyze the disproportionation of superoxide anion free radicals (${\mathrm{O}}_{2}^{o-}$) into molecular oxygen and hydrogen peroxide, thereby reducing the level of OS and inflammation (Yasui & Baba, 2006). Remarkably, some researchers have indicated that exogenous supplementation of SOD can significantly increase endogenous SOD levels and promote the activation of the immune response (Okada et al., 2006; Vouldoukis et al., 2004).

However, these mechanisms are only based on chemical reactions and not on physiological reactions. Based on the above evidence, a reasonable hypothesis can be established, that is, SOD is likely to alleviate hepatic inflammation because of its potent effect on OS. In a previous study, our research group successfully expressed SOD as an extracellular protein of *Bacillus subtilis* and prepared an oral SOD product. This product had good thermal stability and acid resistance, and its activity could reach 22,202 U/g (Dong et al., 2021). In our previous zebrafish experiment, the SOD product significantly inhibited the ROS upregulation caused by deoxynivalenol (DON). To further characterize the hepatic inflammation-alleviating effects of the SOD product, this study established a DON OS model and a hepatic inflammation model in Kunming (KM) mice, investigated the bodyweight changes, alanine transaminase (ALT), aspartate aminotransferase (AST), and several antioxidant indices expression, explored the effects of exogenous SOD on endogenous SOD and proinflammatory cytokine (IL-1*β* and IL-6) expression. Moreover, the effects of exogenous SOD on liver gene expression were also analyzed by transcriptomics to investigate the mechanism of SOD in hepatic inflammation-alleviation, aiming to provide a practical basis for SOD-induced alleviation of OS damage and lay a foundation for future mechanistic analyses.

## Materials and Methods

### Animals

The animals used in this study were adult male specific-pathogen-free (SPF) Kunming (KM) mice that were purchased from the Hubei Center for Disease Control and Prevention.

### Animal care, feeding, housing, and enrichment

All experiments were approved by the Ethics Committee of the Hubei Center for Disease Control and Prevention (NO. 42000600042914) and conducted according to the European Community guidelines (Directive 2010/63/EU). All institutional and national guidelines for the care and use of laboratory animals were followed. All mice were housed in a controlled temperature (22–24 °C) and humidity (40–70%) environment. The light was cycled according to a 12-h light-dark cycle, and the mice were free to eat standard commercial food and distilled water.

### Animal euthanasia

Since it is necessary to collect liver tissue, the mice were euthanized at the end of the experimental period. All the mice were sacrificed using isoflurane. Animals were not excluded from this study and confounders were not controlled.

### DON-induced OS model design

Thirty mice (18–20 g) were randomly divided into five groups (six mice per group), and the experimental period was 15 days. Only the feeding personnel know the detailed grouping. The mice were weighed daily throughout the experiment, and the control group was housed normally. All groups, except the control group, were administered with DON (Meilun Biotechnology Co., Ltd., Dalian, China) *via* gavage to establish a liver OS damage model. The concentration of DON was 0.25 mg/mL, and the gavage dose was 3.2 mL/kg. In addition to the above treatments, SOD prepared with reference to our previous research (Dong et al., 2021) was administered to the low-, medium-, and high-dose groups of mice by gavage at a dose of 5, 10, and 15 U/g, respectively. The treatments provided to each group are presented in Table 1.

**Table 1:** Deoxynivalenol (DON)-induced oxidative stress (OS) animal experimental protocol.

Groups	Experimental protocol
Control group (*n*= 6)	Sterile saline
DON group (*n*= 6)	Sterile saline + DON
Low-dose SOD group (*n*= 6)	Low-dose SOD + DON
Medium-dose SOD group (*n*= 6)	Medium-dose SOD + DON
High-dose SOD group (*n*= 6)	High-dose SOD + DON

**Notes.**

DON Deoxynivalenol SOD superoxide dismutase

### Determination of liver physiological and antioxidant indicators in DON-induced OS mice

At the end of the experimental period, all the mice were sacrificed using isoflurane. Serum was collected from the blood samples by centrifugation at 3,500 × *g* for 20 min. The serum total antioxidant capacity (T-AOC) and ALT, AST, SOD, catalase (CAT), glutathione (GSH), and (glutathione peroxidase) GPX levels were determined using ELISA kits (Nanjing Jiancheng Bioengineering Institute Ltd., Nanjing, China) as per the manufacturer’s instructions. The livers were removed, accurately weighed 0.10 g, and homogenized with pre-cooled sterile saline.

### Hepatic inflammation model design

Thirty mice (14–16 g) were randomly divided into three groups (10 mice per group), and the experimental period was 56 days. Only the feeding personnel know the detailed grouping. The control group was housed normally. All groups, except for the control group, were administered DON *via* gavage and cyclophosphamide (a gift from Zhejiang University) *via* intraperitoneal injection to establish a hepatic inflammation model. The concentration of DON was 0.25 mg/mL, and the gavage dose was 3.2 mL/kg. The dose of cyclophosphamide was 50 mg/kg. In addition to the above treatments, SOD which was prepared with reference to our previous research (Dong et al., 2021), was administered to the SOD group in the feed at a concentration of 3 U/g. The treatments provided to each group are presented in Table 2.

**Table 2:** Hepatic inflammation animal experimental protocol.

Groups	Experimental protocol
Control group (*n*= 10)	Sterile saline
Model group (*n*= 10)	DON + cyclophosphamide + Sterile saline
SOD group (*n*= 10)	DON + cyclophosphamide + SOD

**Notes.**

DON Deoxynivalenol SOD superoxide dismutase

### Determination of serum SOD in mice with hepatic inflammation

At the end of the experimental period, all the mice were sacrificed using isoflurane. Serum was collected from the blood samples by centrifugation at 3,500 × *g* for 20 min. The serum SOD levels were determined using an ELISA kit as per the manufacturer’s instructions.

### Determination of liver IL-1*β* and IL-6 mRNA expression in mice with hepatic inflammation

The method of Li et al. (2020b) was used to measure IL-1*β* and IL-6 mRNA expression in the liver of mice with hepatic inflammation. Briefly, 30 mg of liver tissue was weighed, and Trizol (Vazyme Biotech Co., Ltd., Nanjing, China) was added for homogenization. After 10 min, chloroform was added, and the solution was centrifuged (3,500 × *g*, 20 min, 4 °C) to collect the supernatant. An equal volume of isopropanol was added and the supernatant was collected after centrifugation. Finally, 75% ethanol was added, and the solution was centrifuged again to collect the precipitate. DEPC-treated water was added to resuspended RNA, and the absorbance ratio at 260 nm and 280 nm (A260/280) was assayed to evaluate the concentration of mRNA. For the reverse transcription polymerase chain reaction (RT-PCR) procedure, primers, and sequences, refer to the study by Li et al. (2020b). Finally, the relative quantification of IL-1*β* and IL-6 was analyzed using a comparison with the *β*-actin gene and calculated according to the 2^−ΔΔ*Cq*^ method (Livak & Schmittgen, 2001).

### Transcriptomic analysis of liver in mice with hepatic inflammation

Liver tissue (50 mg) was weighed, and homogenized with Trizol to separate total RNA and determine the concentration, quality, and integrity of the samples. Then, Oligo magnetic beads (New England Biolabs Inc., Ipswich, MA, USA) were used to screen the mRNA with polyA tails from the total RNA and break it into fragments of approximately 300 bp by ion interruption. RNA was reverse transcribed into cDNA with random primers, PCR amplification was performed, and a library was constructed. Next, an Agilent 2100 Bioanalyzer (Agilent Technologies Co., Ltd., Palo Alto, CA, USA) was used to detect the quality and concentration of the library. The library was sequenced using the Illumina next-generation sequencing (NGS) platform (Illumina NovaSeq 6000, Illumina Co., Ltd., San Diego, CA, USA). After filtering the original off-machine data, high-quality sequences obtained was compared with the reference genome, the gene expression was quantified by calculating the fragments per kilobase of exon model per million mapped fragments (FPKM), and differential expression analysis, enrichment analysis, and cluster analysis were performed.

### Statistical analyses

All data was expressed as the mean ± standard deviation (SD). A one-way analysis of variance (ANOVA) was used to analyze the results, and Tukey’s multiple comparison test was used to determine statistical significance. Statistical significance was set at *P* < 0.05.

## Results

### Effect of SOD on the body weight of oxidative stress mice

**Figure 1:**
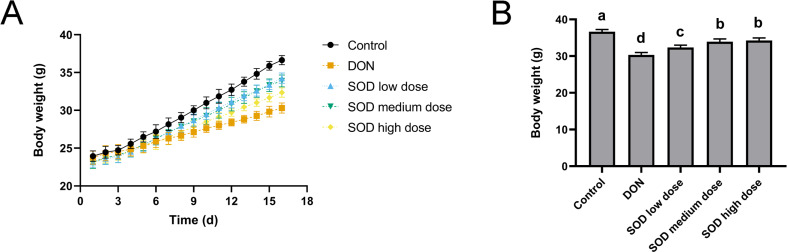
Bodyweight change (*n*= 6). (A) Changes in body weight; (B) body weight at the end of the experiment. Letters a–d indicate statistically significant differences.

As shown in Fig. 1A, the weight gain of the DON group was significantly lower than that of the control group. However, after SOD supplementation, the weight gain was restored. On the last day of the experiment, it was found that the body weight of the DON group was significantly lower than that of the control group (Fig. 1B), but had significantly recovered after SOD supplementation.

### SOD inhibited the increase of ALT and AST levels induced by DON in oxidative stress mice

**Figure 2:**
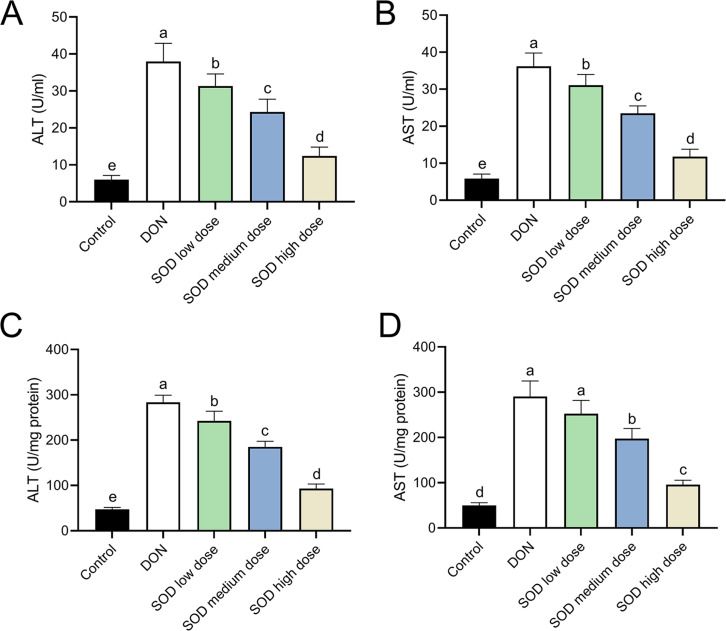
Expression of ALT and AST in deoxynivalenol-induced oxidative stress mice (*n*= 6). The levels of ALT and AST in the serum or liver were determined using the corresponding ELISA kits. (A) ALT in serum; (B) AST in serum; (C) ALT in liver; (D) AST in liver. Letters a–e indicate statistically significant differences. ALT, alanine transaminase; AST, aspartate aminotransferase.

After oral ingestion of DON, serum ALT and AST levels increased rapidly (Figs. 2A and 2B), while liver ALT and AST levels also showed the same trend (Figs. 2C and 2D). After supplementation with exogenous SOD, there was a significant inhibitory effect on ALT and AST levels in both the serum and liver. Moreover, we observed a dose-dependent effect of the SOD product. Low-dose SOD had a slight significant effect, while high-dose SOD significantly reduced ALT and AST levels in the serum and liver (Fig. 2). The effect of medium-dose SOD was between that of the low and high doses. These results suggested that exogenous SOD could potentially alleviate liver OS damage.

### SOD restored the disorder of antioxidant system in oxidative stress mice

**Figure 3:**
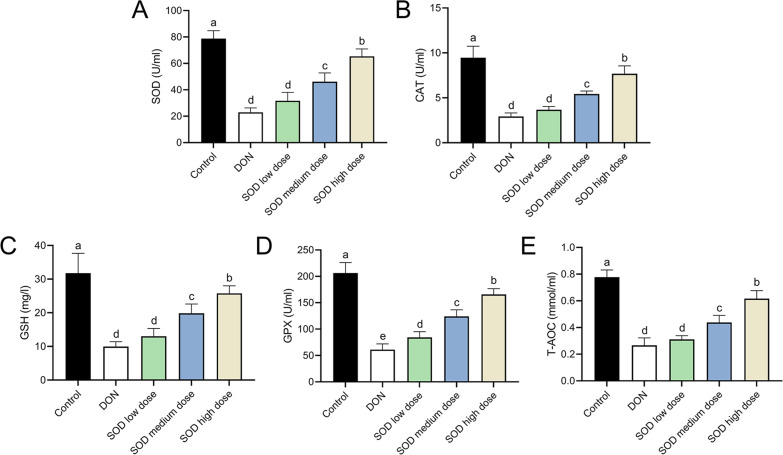
Expression of serum antioxidant indicators in deoxynivalenol induced oxidative stress mice. At the end of the experiment, mice were sacrificed under isoflurane (*n* = 6). Serum was collected from centrifuged blood samples. The (A) SOD, (B) CAT, (C) GSH, (D) GPX, and (E) T-AOC levels in the serum were measured with ELISA assay kits. Letters a–e indicate statistically significant differences. SOD, superoxide dismutase; CAT, catalase; GSH, glutathione, GPX, glutathione peroxidase; T-AOC, total antioxidative capacity.

**Figure 4:**
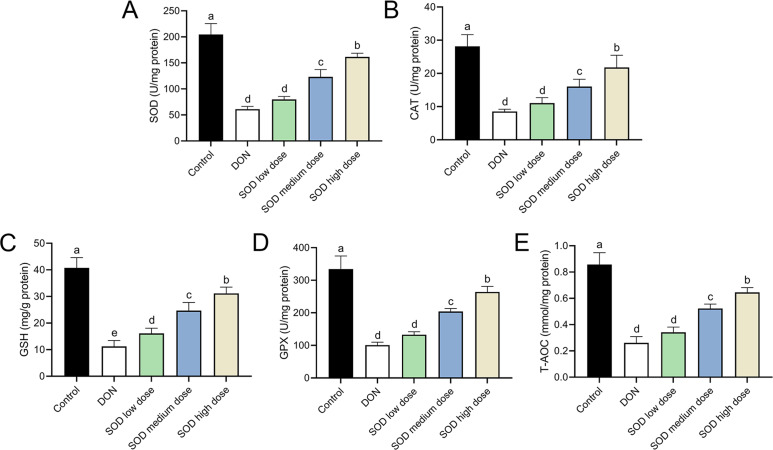
Expression of liver antioxidant indicators in deoxynivalenol induced oxidative stress mice. At the end of the experiment, mice were sacrificed under isoflurane (*n* = 6). Liver tissue was collected. The (A) SOD, (B) CAT, (C) GSH, (D) GPX, and (E) T-AOC levels in the liver were measured with ELISA assay kits. Letters a–e indicate statistically significant differences. SOD, superoxide dismutase; CAT, catalase; GSH, glutathione, GPX, glutathione peroxidase; T-AOC, total antioxidative capacity.

The experimental results indicated that DON had significantly damaged the antioxidant system of mice, as seen by a significant decrease in SOD, CAT, GSH, and GPX expression. There was also a significant decrease in T-AOC (Figs. 3 and 4). Meanwhile, oral administration of the SOD product significantly restored the expression of SOD, CAT, GSH, and GPX, and increased T-AOC (Figs. 3 and 4). As with the effects on ALT and AST levels, exogenous SOD also had a dose-dependent effect in restoring the antioxidant indicators. High-dose SOD showed the best OS recovery effect.

### SOD alleviated OS damage in the hepatic inflammation

**Figure 5:**
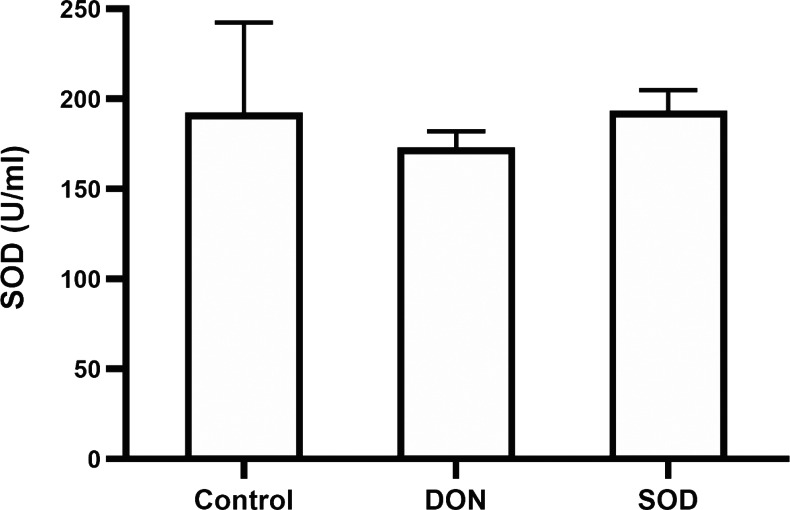
Expression of serum SOD in mice with hepatic inflammation (*n*= 3). At the end of the experiment, mice were sacrificed under isoflurane. Serum was collected from centrifuged blood samples. The content of SOD in the serum was determined using the corresponding ELISA kits. SOD, superoxide dismutase.

Similar to the effect of DON in the OS model, the combination of DON and cyclophosphamide reduced serum SOD levels of mice with hepatic inflammation. This change was restored by exogenous SOD supplementation, indicating that the SOD product effectively alleviated OS damage in the hepatic inflammation mouse model (Fig. 5).

### SOD inhibited the expression of pro-inflammatory cytokines in the hepatic inflammation

**Figure 6:**
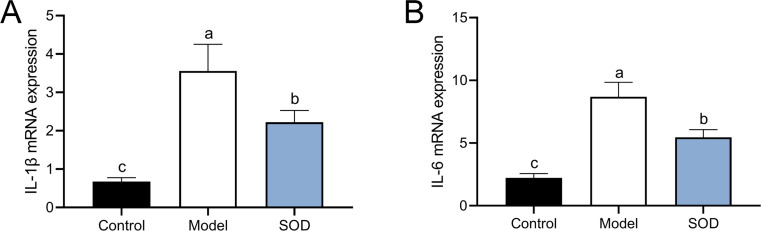
MRNA expression of liver IL-1 *β* andIL-6 in mice with hepatic inflammation (*n*= 5). At the end of the experiment, mice were sacrificed under isoflurane. Liver tissue was collected. 30 mg of liver tissue was weighed and homogenized. Then chloroform, isopropanol, and 75% ethanol were added to collect the precipitate. (A) IL-1 *β* and (B) IL-6 mRNA expression in the liver was measured by the reverse transcription polymerase chain reaction. Letters a–c indicate statistically significant differences.

The combination of DON and cyclophosphamide significantly increased IL-1*β* and IL-6 mRNA expression in the livers of mice with hepatic inflammation (Fig. 6). However, exogenous SOD significantly inhibited the mRNA expression of these proinflammatory cytokines, which proves the alleviating effects of SOD on hepatic inflammation (Fig. 6).

### SOD regulated liver transcriptomics in the hepatic inflammation

**Figure 7:**
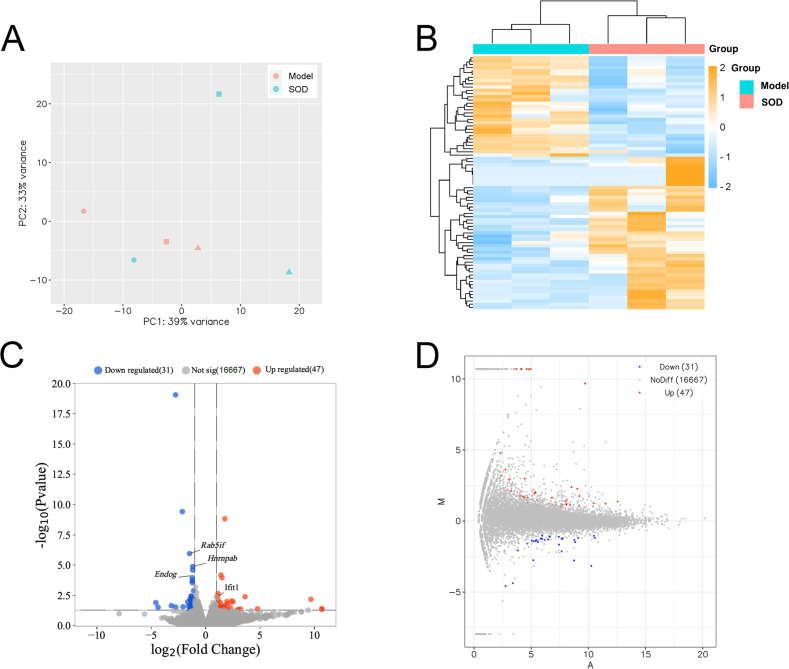
Liver transcriptome analysis in mice with hepatic inflammation (*n*= 3). Liver tissue was weighed, and homogenized to separate total RNA. The mRNA with polyA tails were screened from the total RNA breaked into fragments. RNA was reverse transcribed into cDNA and constructed a library. Then the library was sequenced and analysed. (A) Principal component analysis; (B) Cluster analysis of the differentially expressed genes, and the colored scale bar indicates the normalized value of expression level of differentially expressed genes processed by log_2_ (FPKM + 1) protocol; (C) Volcano plot; (D) M-versus-A plot.

Principal component analysis was conducted based on expression, using the R language DESeq software package (Fig. 7A). Cluster analysis classified genes with high expression levels between samples into one category, using the R language Pheatmap software package (Fig. 7B). The results indicated that the model and SOD groups could be clustered separately, which suggested that exogenous SOD had significant hepatic inflammation-alleviating effects in mice.

The volcano plot and M-versus-A plot of differentially expressed genes was prepared using the R language ggplot2 software package, and the results are shown in Figs. 7C and 7D, respectively. The results indicated that exogenous SOD caused downregulation of 31 genes and the upregulation of 47 genes. The filtering criteria of differentially expressed genes was |log_2_ Fold change|> 1 and *P* < 0.05.

**Figure 8:**
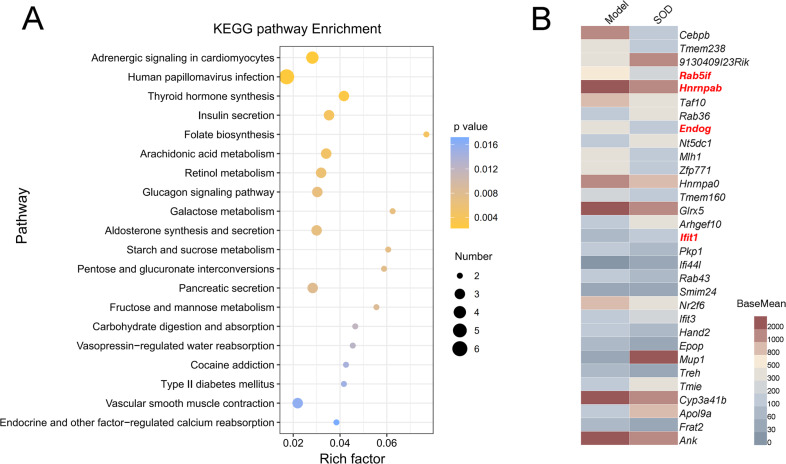
Analysis of differential expression of liver genes in mice with hepatic inflammation (*n*= 3). The library was sequenced and high-quality sequences obtained was compared with the reference genome. Then the gene expression was calculated, and differential expression analysis, enrichment analysis, and cluster analysis were performed. (A) Kyoto Encyclopedia of Genes and Genomes (KEGG) analysis; (B) Heat map of differential gene expression (*P* < 0.01). The genes that were significantly correlated with hepatic inflammation are shown in red color names. BaseMean indicates the average value of the normalized count values, dividing by size factors, taken over all samples. The BaseMean values were obtained from Deseq2 differential expression analysis dataset.

The gene expression of different groups of mice with hepatic inflammation was quantified and compared with the Kyoto Encyclopedia of Genes and Genomes (KEGG) database. Preliminary results are shown in Fig. 8A. Exogenous SOD mainly changed the endocrine- and carbohydrate metabolism-related pathways in mice; such as adrenergic signaling, glucagon signaling, aldosterone synthesis and secretion, retinol metabolism, arachidonic acid metabolism, *etc*.

The genes with differential expression were analyzed for statistical significance, those with *P* < 0.01 were selected, and the results are shown in Fig. 8B. Oral supplementation of exogenous SOD significantly inhibited the expression of *Rab5if*, *Hnrnpab*, and *Endog*, but increased the expression of *Ifit1*.

## Discussion

This study aimed to verify the OS-alleviating effects of a highly viable SOD oral product that was prepared by our research group in a previous study (Dong et al., 2021), and to investigate its effects and mechanisms in alleviating hepatic inflammation. Therefore, we established a mouse liver OS damage model with DON and a mouse hepatic inflammation model with a combination of DON and cyclophosphamide. We characterized the effects of exogenous SOD in restoring the antioxidant system in mice with liver OS damage, explored its effects in alleviating hepatic inflammation, and finally performed a preliminary analysis of its mechanism using transcriptomics.

Accumulating evidence suggests that the occurrence of OS is correlated with dietary intake. The types and quantities of nutrients significantly impact the development of OS, and an excessive or lack of dietary intake can induce metabolic OS (Tan, Norhaizan & Liew, 2018). DON is a mycotoxin commonly found in grains (Bonnet et al., 2012). Several studies have indicated that DON induces ROS production and causes OS-related damage to the body (Mishra et al., 2014; Ren et al., 2020; Wu et al., 2014). However, in living organisms, only the liver can convert DON into DON-glucuronic acid by glucuronyltransferase (Peng et al., 2017). Therefore, the liver is generally regarded as the target organ for DON-induced OS damage. We first measured the changes in the body weight of the mice and the physiological indicators of the liver. In animal models, body weight is often used to reflect the severity of the disease. ALT and AST are widespread enzymes in the cytoplasm of hepatocytes and are clinically considered as reliable and sensitive indicators for diagnosing liver diseases (Lei et al., 2020). Compared with the control group, the body weight of mice orally taking DON was lower, which indicated that the growth of mice was inhibited. However, the expression of ALT and AST in the liver and blood increased significantly. These physiological changes are consistent with previous studies on the toxicity of DON (Bonnet et al., 2012), indicating that the liver OS damage model was successfully established. Furthermore, we characterized the damage to the antioxidant system in mice. The antioxidant system of an organism can actively alleviate OS symptoms. SOD is present in all living organisms and is the most frontline substance against OS damage (Griess et al., 2017). CAT is an enzyme mainly found in the peroxisomes of mammalian cells and is a coenzyme of other antioxidant enzymes (Tehrani & Moosavi-Movahedi, 2018). The sulfhydryl group of GSH participates in reduction reactions and is thus regarded as the most critical low-molecular-weight antioxidant synthesized in animal cells (Forman, Zhang & Rinna, 2009). GPX is generally subjected to antioxidant translation using GSH as a reducing agent (Brigelius-Flohé & Maiorino, 2013). We found that the levels of SOD, CAT, GSH, and GPX in the blood and liver significantly decreased after DON administration. This suggested that DON caused significant damage to the antioxidant system in mice, consistent with liver OS damage caused by other toxins (Irshad et al., 2021; Zhai et al., 2014). Therefore, we believe that the damage caused by DON in the liver should be standard OS-related damage.

Next, we investigated the OS damage-alleviating effects of exogenous SOD. We found that SOD positively affected body weight, liver physiological indicators, and antioxidant systems in the OS damage model. This indicated that exogenous SOD supplementation could significantly restore the antioxidant system in mice, thereby protecting mice from OS damage. Moreover, the conclusion that this exogenous SOD supplementation induces the enhancement of the systemic antioxidant system has been previously reported (Carillon et al., 2013). In fact, one of the functions of CAT and GPX in organisms is to remove the toxic H_2_O_2_ produced by SOD ${\mathrm{O}}_{2}^{o-}$ disproportionation (Carillon et al., 2013). Therefore, the supplementation of exogenous SOD is likely to increase the H_2_O_2_ concentration in the body, further increasing the expression of CAT and GPX. This is actually a defense mechanism. However, there was a dose-dependent effect of SOD in alleviating OS damage. High-dose SOD had the most potent alleviating effect, whereas low-dose SOD had a weak effect. The dose-dependent effect of SOD has been reported in previous studies. For example, Bernier et al. found that the dose–response curve of SOD in a rat cardiac reperfusion model formed an asymmetric U-shape. As the dose increased, the alleviating effects of SOD gradually increased. However, when the SOD concentration in the perfusate reached 120,000 U/L, the alleviating effects were significantly reduced (Bernier, Manning & Hearse, 1989). Similarly, Nelson, Bose & McCord (1994) reported that the alleviating effects of SOD showed a bell-shaped dose–response curve. The alleviating effects increased as the SOD dose increased. However, at a dose of 50 mg/L, SOD loses its protective ability and severely exacerbates damage. These studies indicate that a very high dose of SOD is toxic. The dose-dependent effect of SOD may be related to the characteristics of superoxide anion free radicals. In addition to causing OS, superoxide anion free radicals also act as terminators for lipid peroxidation (Nelson, Bose & McCord, 1994). Excessive scavenging of free radicals will likely, and paradoxically, increase lipid peroxidation. Therefore, while high doses of SOD are toxic, the SOD dose used in this study was still in the low-dose range. Even in the high-dose SOD group, the SOD dose did not reach the inflection point of the SOD response curve. Hence, for the various indicators of this experiment, the alleviating effects of SOD increased as dose increased.

As noted above, exogenous SOD alleviated OS damage in mice. However, previous investigations have also found that hepatic inflammation is inseparable from OS. To be precise, there is an interactive relationship between OS and liver inflammation. In the OS state, the liver usually suffers oxidative damage, which further triggers the activation of inflammatory signaling pathways and apoptosis (Ávila Escalante et al., 2020). On the contrary, in an inflammatory state, the metabolic balance of the liver is disrupted, leading to accelerated production of ROS (Alfadda & Sallam, 2012). This suggests that targeted intervention for OS is highly likely to help alleviate the accompanying liver inflammation. Therefore, we established a hepatic inflammation model to verify this hypothesis and to explore the possible mechanism of the hepatic inflammation-alleviating effects of exogenous SOD.

As a widely used anti-tumor drug, cyclophosphamide has a remarkable effect. However, due to the hydroxylation of the liver, cyclophosphamide can be converted into 4-hydroxycyclophosphamide, which indicates potential liver metabolic toxicity and can cause hepatic inflammation (Voelcker, 2020). Moreover, the current study found that cyclophosphamide increased the iron level and ferritin expression in the liver, thus inhibiting the production of red blood cells and the synthesis of hemoglobin, finally leading to a reduction of iron utilization and the occurrence of OS (Sheng et al., 2020). Therefore, we established a mouse model of hepatic inflammation using DON combined with cyclophosphamide. Within the model, we found that the level of SOD in the blood of mice was reduced and hepatic inflammation had occurred (a significant increase in the expression of proinflammatory cytokines). However, exogenous SOD significantly restored these abnormalities. This proved that exogenous SOD could alleviate hepatic inflammation, and this may have been related to the alleviation of OS. The effects of SOD on alleviating OS- and inflammation damage concomitantly has been reported in the previous study. Such as, Vouldoukis et al. (2004) have demonstrated the improvement in the antioxidant system and proinflammatory cytokine (tumor necrosis factor-alpha, TNF-*α*) induced by the Cucumis melo extracts rich in SOD in mice interferon- *γ* inflammation model. Our results are similar to them. However, the mechanism remains to be analyzed. Hence, to further analyze the hepatic inflammation-alleviating mechanism of the SOD product, we performed a transcriptomic analysis of gene expression in the mouse liver. The results showed that the established model experienced downregulation of 31 genes and upregulation of 47 genes. Among them, *Rab5if*, *Hnrnpab*, *Endog*, and *Ifit1* were significantly correlated with hepatic inflammation.

*Rab5if* is a long-chain, non-coding RNA RAB5 interacting factor. It is highly expressed in patients with liver cancer and is significantly related to poor prognosis, and mediates liver cancer and apoptosis by regulating LGR5-mediated *β*-catenin and c-Myc signaling (Koo et al., 2019). *Hnrnpab* is a gene encoding for a heterogeneous ribonucleoprotein that is highly expressed in tumor cells, and participates in several cancer-related pathways; such as G2/M checkpoints, DNA repair, and IL6/JAK/STAT3 signaling (Li et al., 2020a). *Endog* encodes for endonuclease g, which is an apoptotic effector, which has been significantly correlated with OS damage and apoptosis of liver cells (Kapoor & Kakkar, 2014). *Ifit1* encodes for an interferon-inducing protein with tetratricopeptide repeat 1. The exaggerated expression of *Ifit1* in liver cells can inhibit c-Jun N-terminal kinase signal transduction by binding to the Axin protein and impairing its activity, thereby inhibiting TNF-*α*-mediated hepatocyte apoptosis, and ultimately alleviating hepatic inflammation (Chang et al., 2015). Therefore, we speculate that the SOD product significantly inhibited the *Endog* expression and restore the antioxidant system function, thereby inhibiting OS damage, and regulating hepatocyte apoptosis through the combined regulation of *Rab5if*, *Hnrnpab*, and *Ifit1* expression, ultimately alleviating hepatic inflammation.

## Conclusion

In summary, this study aimed to verify the alleviating effects of the SOD products prepared in previous research on OS damage, explore the alleviating effects of this product on hepatic inflammation, and analyze the correlation between the OS- and hepatic inflammation-alleviating effects. We found that this exogenous SOD significantly alleviated liver OS damage and hepatic inflammation in the established animal models. Transcriptomic analysis indicated that the hepatic inflammation-alleviating effects of the SOD product was most likely mediated by regulating the expression of *Rab5if*, *Hnrnpab*, *Endog*, *Ifit1*, which alleviated OS and liver cell apoptosis.

##  Supplemental Information

10.7717/peerj.15829/supp-1 Data S1 Raw Data

10.7717/peerj.15829/supp-2 Supplemental Information 2 ARRIVE guidelines 2.0

## Additional Information and Declarations

### Data Availability

The following information was supplied regarding data availability: The raw data is available in the Supplemental Files.
